# Characterization of a novel *covS* SNP identified in Australian group A *Streptococcus* isolates derived from the M1_UK_ lineage

**DOI:** 10.1128/mbio.03366-24

**Published:** 2024-12-17

**Authors:** Johanna Richter, Amanda J. Cork, Yvette Ong, Nadia Keller, Andrew J. Hayes, Mark A. Schembri, Amy V. Jennison, Mark R. Davies, Kate Schroder, Mark J. Walker, Stephan Brouwer

**Affiliations:** 1 Institute for Molecular Bioscience, Australian Infectious Diseases Research Centre, The University of Queensland, Brisbane, Queensland, Australia; 2 School of Chemistry and Molecular Biosciences, The University of Queensland, Brisbane, Queensland, Australia; 3 Department of Microbiology and Immunology at the Peter Doherty Institute for Infection and Immunity, The University of Melbourne, Melbourne, Australia; 4 Public and Environmental Health, Pathology Queensland, Queensland Health, Coopers Plains, Queensland, Australia; 5 Institute for Molecular Bioscience, Centre for Inflammation and Disease Research, The University of Queensland, Brisbane, Queensland, Australia; Universite de Geneve, Geneva, Switzerland

**Keywords:** *Streptococcus pyogenes*, two-component system, CovRS, SNP, transcriptional regulation, SLO, SpeB, inflammation, interleukin-1β

## Abstract

Group A *Streptococcus* (GAS) is a human-adapted pathogen responsible for a variety of diseases. The GAS M1_UK_ lineage has contributed significantly to the recently reported increases in scarlet fever and invasive infections. However, the basis for its evolutionary success is not yet fully understood. During the transition to systemic disease, the M1 serotype is known to give rise to spontaneous mutations in the control of virulence two-component regulatory system (CovRS) that confer a fitness advantage during invasive infections. Mutations that inactivate CovS function result in the de-repression of key GAS virulence factors such as streptolysin O (SLO), a pore-forming toxin and major trigger of inflammasome/interleukin-1β-dependent inflammation. Conversely, expression of the streptococcal cysteine protease SpeB, which is required during initial stages of colonization and onset of invasive disease, is typically lost in such mutants. In this study, we identified and characterized a novel *covS* single nucleotide polymorphism detected in three separate invasive M1_UK_ isolates. The resulting CovS^Ala318Val^ mutation caused a significant upregulation of SLO resulting in increased inflammasome activation in human THP-1 macrophages, indicating an enhanced inflammatory potential. Surprisingly, SpeB production was unaffected. Site-directed mutagenesis was performed to assess the impact of this mutation on virulence and global gene expression. We found that the CovS^Ala318Val^ mutation led to subtle, virulence-specific changes of the CovRS regulon compared to previously characterized *covS* mutations, highlighting an unappreciated level of complexity in CovRS-dependent gene regulation. Continued longitudinal surveillance is warranted to determine whether this novel *covS* mutation will expand in the M1_UK_ lineage.

**IMPORTANCE:**

The M1_UK_ lineage of GAS has contributed to a recent global upsurge in scarlet fever and invasive infections. Understanding how GAS can become more virulent is critical for infection control and identifying new treatment approaches. The two-component CovRS system, comprising the sensor kinase CovS and transcription factor CovR, is a central regulator of GAS virulence genes. In the M1 serotype, *covRS* mutations are associated with an invasive phenotype. Such mutations have not been fully characterized in the M1_UK_ lineage. This study identified a novel *covS* mutation in invasive Australian M1_UK_ isolates that resulted in a more nuanced virulence gene regulation compared to previously characterized *covS* mutations. A representative isolate displayed upregulated SLO production and triggered amplified interleukin-1β secretion in infected human macrophages, indicating an enhanced inflammatory potential. These findings underscore the need for comprehensive analyses of *covRS* mutants to fully elucidate their contribution to M1_UK_ virulence and persistence.

## INTRODUCTION

*Streptococcus pyogenes* (group A *Streptococcus* [GAS]) is an important human-adapted pathogen that causes a wide spectrum of diseases in humans, ranging from mild infections of epithelial or mucosal tissues such as pharyngitis (strep throat) and impetigo to severe invasive diseases including bacteremia and necrotizing fasciitis. Repeated and untreated GAS infections can result in immune response-mediated pathologies that can progress to chronic autoimmune conditions, such as rheumatic heart disease (RHD) (1). RHD accounts for the majority of GAS-related morbidity and mortality worldwide (2). An estimated 616 million infections and more than 500,000 deaths per year are attributed to GAS, which predominantly affect young children and people living in countries with poor healthcare infrastructure (2).

The M1 serotype of GAS underwent several horizontal gene transfer events in the mid-1980s, which resulted in the emergence of the hyperinvasive M1T1 clone (referred to as “M1_global_” in this study). Clones isolated after 1988 had acquired novel prophages carrying the superantigen allele *speA2* and the DNase gene *sda1*/*sdaD2* (3, 4). In addition, genetic recombination equipped these clones with a high-expression *nga-ifs-slo* operon encoding a NAD^+^-glycohydrolase (NADase) with an associated endogenous inhibitor and the cytolytic toxin streptolysin O (SLO) that has been associated with increased virulence in M1_global_ and certain acapsular GAS isolates (3, 5–7). M1_global_ became the major driver of invasive GAS infections in high-income countries (1, 3, 8, 9). However, since 2008, a new M1 sublineage, referred to as M1_UK_, has emerged and significantly contributed to scarlet fever outbreaks in the UK and a surge in invasive infections in various countries (10–21). M1_UK_ differs from M1_global_ by the presence of 27 core genome single-nucleotide polymorphisms (SNPs) (10, 22). Although the transcriptional heterogeneity between M1_global_ and M1_UK_ is reportedly low (22, 23), the M1_UK_ lineage is characterized by increased *in vitro* expression of the SpeA superantigen caused by a single SNP in the *ssrA* gene (encoding a transfer-messenger RNA) that results in *ssrA* terminator read-through and subsequent higher expression of the *speA* gene located downstream of *ssrA* (22). However, the specific mechanisms driving the clonal success of M1_UK_ remain incompletely understood.

The control of virulence two-component regulatory system (CovRS), which consists of the sensor kinase CovS and the transcription factor CovR, regulates up to 15% of the GAS genome (24–27) and coordinates the response to stress conditions, such as limited nutrient availability and host-pathogen interaction (26, 28–32). During homeostasis and in non-invasive settings, CovS activity maintains the DNA-bound state of CovR (33) to suppress the expression of numerous virulence-associated genes (34), including those encoding SLO (*slo*), streptokinase (*ska*), and the hyaluronic acid capsule (*hasA*) (24–27). Mutations in *covS* that can spontaneously occur during invasive disease progression of M1_global_ GAS typically result in the upregulation of these virulence factors (25, 35, 36). Additionally, inactivating *covS* mutations often cause the loss of the broad-spectrum protease SpeB (25, 35, 37–39), thus preventing the SpeB-mediated degradation of other GAS virulence factors (40, 41). Combined, these events confer an invasive phenotype with enhanced immune evasion and a decreased ability to colonize nasopharyngeal tissues (35–38, 42–45). A recent publication noted that the frequency of *covRS* mutations was lower in M1_UK_ compared to M1_global_ invasive isolates collected in the UK between 2014 and 2023 (20); however, it is currently unknown whether this trend generally applies to M1_UK_. CovRS mutants derived from the M1_UK_ lineage have not yet been fully characterized.

In addition to CovRS regulation, the progression of invasive GAS infections is also controlled by host factors, such as the pro-inflammatory cytokine interleukin-1β (IL-1β) that is produced by the inflammasome pathway (46). IL-1β signaling plays a crucial but complex role in GAS disease. It is required to prevent the systemic spread of GAS (47, 48), but once invasive infection is established, high levels of IL-1β correlate with increased disease severity (49, 50). Of note, CovRS mutants demonstrate an enhanced ability to survive the consequences of IL-1β signaling, such as neutrophil-mediated clearance (36, 51). In addition, IL-1β secretion was shown to promote GAS colonization of the nasopharynx (52). Accordingly, the inflammasome/IL-1β axis has been suggested as a potential target for therapeutic intervention (47, 50, 52–54). The streptolysins SLO and SLS secreted by GAS have been shown to trigger IL-1β production in a variety of cell types (55–59) and are the major contributors to inflammasome activation in human macrophages (57). The extent of IL-1β signaling stimulated by M1_UK_ isolates has not yet been investigated.

In this study, we identified a novel *covS* SNP in a subset of invasive Australian M1_UK_ isolates that altered the transcription of genes in the CovRS regulon to a smaller degree than previously characterized *covS* mutations. Isolates carrying this mutation expressed significantly higher levels of SLO and stimulated increased inflammasome signaling in human macrophages, indicating enhanced inflammatory potential. However, production of the streptococcal cysteine protease SpeB was unaffected by this mutation. These findings provide new insights into the virulence strategies of invasive M1_UK_ isolates.

## RESULTS

### Additional single-nucleotide polymorphisms in a subset of Australian M1_UK_ isolates

In a previous large-scale genomic analysis of Australian invasive GAS M1 isolates, 10 M1_UK_ isolates were found to possess between 8 and 17 SNPs in addition to the 27 lineage-defining SNPs (Table 1) (10, 22). Among these mutations, seven SNPs were synonymous, and two SNPs were located in intergenic regions. A further two SNPs created preliminary stop codons in *purC*, which is involved in purine biosynthesis (60), and H7X56_07280, which encodes a phage-related DNA primase. The remaining six SNPs caused non-synonymous changes in the transcriptional regulators RofA (RofA^Ile279Thr^) and RocA (RocA^Asp397Gly^), the sensor kinase CovS (CovS^Ala318Val^)*,* as well as in three uncharacterized genes. Of note, the RofA^Ile279Thr^ mutation is distinct from the three RofA mutations already present in M1_UK_, which were previously shown not to have a conserved, phenotypic impact (61).

**TABLE 1:** Overview of additional SNPs found in a subset of Australian M1_UK_ isolates^*a*^

Nucleotide position	Gene	Nucleotide change	Amino acid change	Isolates									
				SP1450	SP1466	M1800976	M14002397	M13006651	M13006969	M14000714	M14007357	GCA_900995035	GCA_900995115
35,938	*purC*	C > A	Ser53* (stop gained)	X	X	X	X	X	X	X	X	X	X
116,047	*rofA*	T > C	Ile279Thr	X	X	X	X	X	X	X	X	X	X
318,608	*covS*	C > T	Ala318Val	X	X	X							
372,461	*scpC*	A > C	Ile1068 synonymous	X	X	X	X	X	X	X	X	X	X
710,002	*mvaK2*	A > G	Pro130 synonymous	X	X	X	X	X	X	X	X	X	X
718,627	H7X56_03610	G > A	Glu142 synonymous	X	X	X	X	X	X	X	X	X	X
762,893	Intergenic region	G > A	–	X	X	X	X	X	X	X	X	X	X
983,947	H7X56_04920	dupl. A	Thr52 frameshift	X	X	X	X	X	X				
1,017,939	H7X56_05085	C > T	Asn482 synonymous	X	X	X	X	X	X	X	X	X	X
1,188,262	Intergenic region	G > A	–	X									
1,193,010	*recT*	G > A	Lys134 synonymous	X									
1,209,205	H7X56_06155	G > A	Ala202 synonymous	X	X								
1,302,773	*rocA*	A > G	Asp397Gly	X	X								
1,439,544	H7X56_07280	C > T	Gln3* (stop gained)	X	X	X	X	X	X	X	X		
1,524,813	*endoS*	C > T	Asp366 synonymous	X	X	X	X	X	X	X	X	X	X
1,696,650	H7X56_08660	del. A	Met170 frameshift	X	X	X							
1,764,502	H7X56_08955	G > T	Lys221Asn	X	X	X	X	X	X	X	X		

^*a*^ SNPs are identified by their nucleotide positions in comparison with the SP1380 (M1_UK_) reference genome (National Center for Biotechnology Information accession number CP060269.1). X indicates that the SNP is present in the listed isolate. – indicates the absence of an amino acid change due to the intergenic location of the SNP.

Given that RofA, RocA, and CovS have known functions in the transcriptional regulation of GAS virulence factor gene expression (24–27, 62–64), we investigated whether one or more of the additional mutations found in the Australian M1_UK_ isolates alter the GAS virulence profile, using SP1450 and SP1466 as two representative isolates.

### A novel SNP in *covS* drives increased SLO expression in Australian M1_UK_ isolates

**Fig 1:**
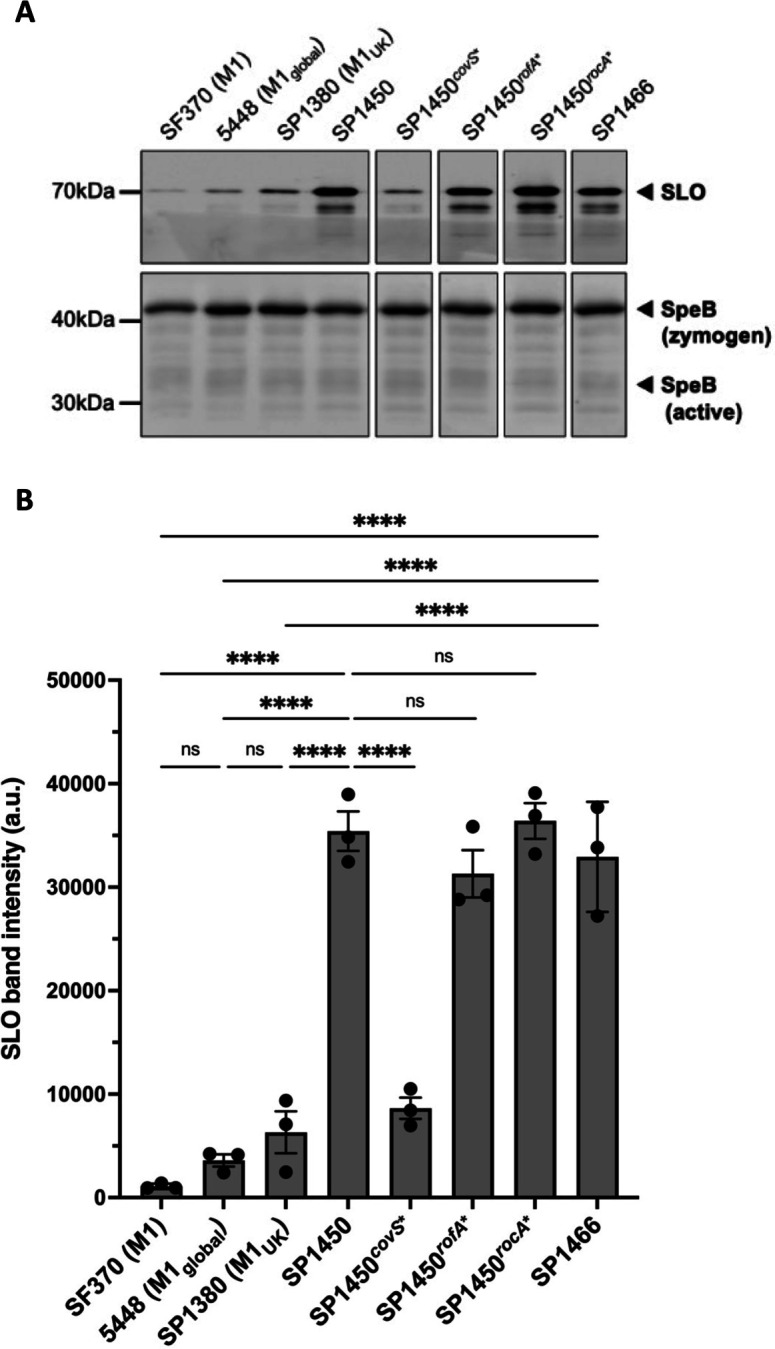
Protein expression of SLO and SpeB in M1 GAS strains and SP1450 isogenic mutants. All strains were grown in Todd-Hewitt broth supplemented with 1% yeast extract to the late exponential growth phase (optical density at 600 nm = 0.8). Supernatant protein was purified using trichloroacetic acid precipitation. Expression of SLO and SpeB was visualized via immunoblotting. (**A**) Representative Western blot of *n* = 3 biological repeats; upper panel shows SLO; lower panel shows SpeB. Gaps between lanes indicate that additional samples have been cropped out of the full blot image (see Fig. S2). (**B**) Quantification of SLO expression of *n* = 3 immunoblots, which was performed using ImageJ. Error bars represent SEM. Significance was analyzed by Tukey one-way analysis of variance. ****P < 0.0001. ns, not significant.

First, we compared protein expression levels of two major GAS virulence factors, SLO and SpeB, in the Australian M1_UK_ isolates SP1450 and SP1466, which both harbor the additional SNPs in *rofA*, *covS*, and *rocA*, to representative GAS strains of the pre-1988 M1 genotype (SF370) (65), the post-1988 M1_global_ genotype (5448) (66), and the contemporary M1_UK_ genotype (SP1380) (22). SLO protein abundance in culture supernatants of SP1450 and SP1466 was significantly increased (~6-fold) compared to the other M1 GAS strains (Fig. 1A and B), while SpeB levels remained unchanged (Fig. 1A; Fig. S1), despite the presence of the CovS^Ala318Val^ mutation in SP1450 and SP1466.

To identify which of the mutations in CovS, RofA, and RocA is responsible for the increased SLO expression in SP1450 and SP1466, we generated a set of isogenic mutants in SP1450, where the SNPs in the respective genes were individually corrected to reflect the M1_global_ genotype (SP1450^covS*^, SP1450^rofA*^, and SP1450^rocA*^). SLO protein levels remained significantly increased in SP1450^rofA*^ and SP1450^rocA^^*^, while correction of the *covS* SNP significantly reduced SLO production in SP1450^covS^^*^ to a level comparable to that of the M1, M1_global_, and M1_UK_ reference strains (Fig. 1A and B).

**Fig 2:**
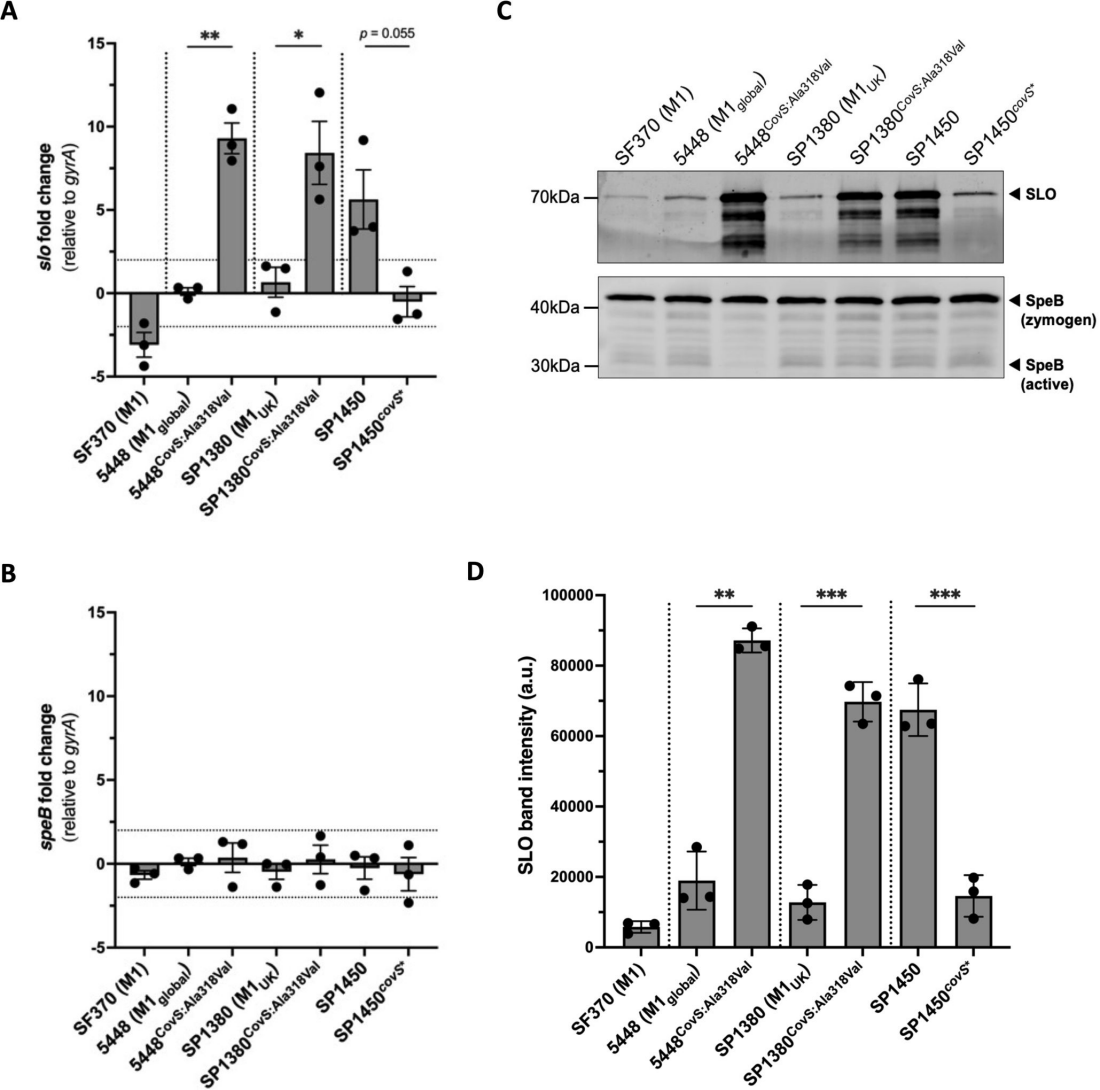
CovS^Ala318Val^ increases *slo* expression in M1 GAS. To account for possible RNA degradation at later growth stages, strains were grown in Todd-Hewitt broth supplemented with 1% yeast liquid culture to the mid-exponential growth phase (optical density at 600 nm = 0.4). Quantified RNA transcripts of *slo* (**A**) and *speB* (**B**), relative to the *gyrA* gene, were normalized to 5448. Precipitated preparations of total culture supernatant protein were probed for SLO and SpeB by immunoblotting. (**C**) Representative Western blot of *n* = 3 biological repeats; upper panel shows SLO, and lower panel shows SpeB. (**D**) SLO protein expression in *n* = 3 replicates was quantified with ImageJ. All error bars represent SEM. Significance was analyzed by unpaired Welch *t*-test comparing wild-type strains and respective isogenic mutants. *P < 0.05, **P < 0.01, ***P < 0.001.

To validate this finding, we introduced the *covS* SNP, resulting in the CovS^Ala318Val^ mutation into the M1_global_ and M1_UK_ genetic backgrounds to create 5448^CovS:Ala318Val^ and SP1380^CovS:Ala318Val^, respectively, and analyzed RNA transcript levels of *slo* and *speB* in both isogenic mutants, as well as in SP1450 and SP1450^covS*^ (Fig. 2A). A significant increase (~8.5-fold) in *slo* transcripts was detected in 5448^CovS:Ala318Val^ and SP1380^CovS:Ala318Val^ compared to the respective parental strains. By contrast, repair of the CovS^Ala318Val^ mutation in SP1450 (SP1450^covS*^) reduced *slo* transcription levels (not statistically significant). No changes in *speB* transcript levels were detected in any of the mutant strains (Fig. 2B), confirming previous observations. The parallel measurement of protein abundances revealed that SLO production was increased approximately 5-fold in 5448^CovS:Ala318Val^ and SP1380^CovS:Ala318Val^, similar to the levels produced by SP1450 (Fig. 2C and D). Conversely, SLO levels in SP1450^covS*^ were significantly reduced and comparable to the amounts secreted by the other M1 strains. This differential expression pattern of SLO was not affected by the presence of LL-37 (data not shown), indicating that the observed effects of CovS^Ala318Val^ likely have physiological relevance. In addition, neither the presence nor the absence of CovS^Ala318Val^ affected SpeB protein levels (Fig. 2C; Fig. S1). Similar growth behavior of all strains confirmed that the observed effects of CovS^Ala318Val^ were not due to growth differences between isolates and mutants (Fig. S2). Taken together, these results suggest that the CovS^Ala318Val^ mutation detected in a subset of Australian M1_UK_ isolates drives increased SLO expression.

Numerous mutations in *covS* have been reported in the literature, but only a few have been functionally characterized (25, 39, 67, 68). To determine the novelty of the *covS*^C953T^ SNP (resulting in CovS^Ala318Val^), a tBLASTn search was performed against 109,874,994 nucleotide sequences submitted to the National Center for Biotechnology Information nucleotide database (accessed on 23 October 2024 with the search filter “*Streptococcus pyogenes* [taxid1314]”). Using sequences spanning a region of 15 amino acids up- and downstream of position 318 in CovS, the search identified no non-synonymous *covS* mutations that would result in the Ala318Val substitution. In addition, a direct protein BLAST search also did not yield any matching amino acid sequence in isolates other than those described in this study, suggesting that this mutation has not been previously identified.

### Impact of the CovS^Ala318Val^ mutation on the CovRS regulon

The role of the CovRS two-component system in gene regulation and virulence of GAS is well established (1, 24, 33, 34, 69–71). To investigate the impact of the novel CovS^Ala318Val^ mutation on global gene expression, we performed RNA sequencing analysis of the strain pairs 5448/5448^CovS:Ala318Val^ and SP1380/SP1380^CovS:Ala318Val^, grown to the mid-exponential growth phase *in vitro*.

**Fig 3:**
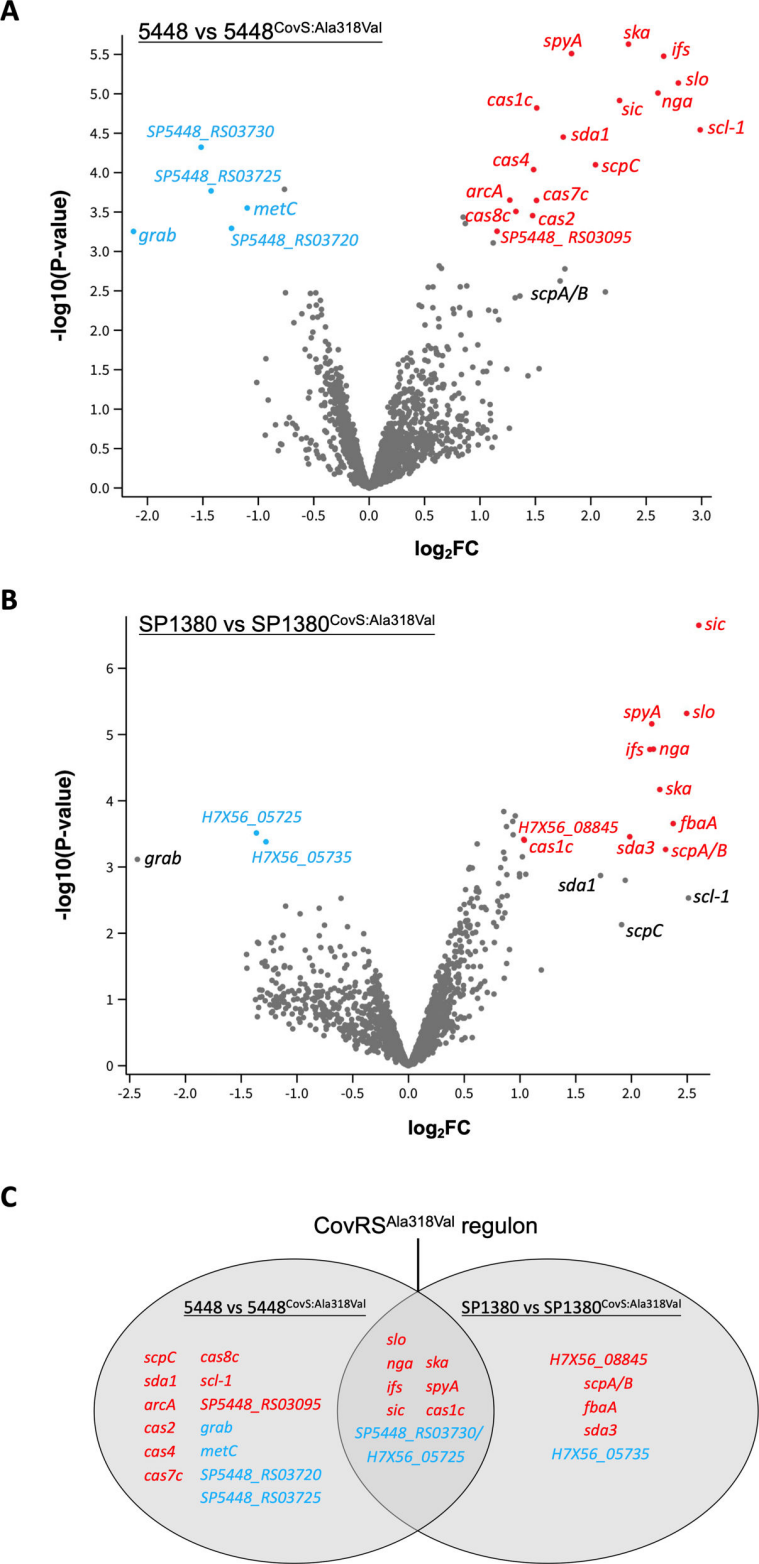
RNA sequencing analysis investigating the impact of CovS^Ala318Val^ on global gene transcription in 5448 and SP1380. (**A** and **B**) Volcano plots highlighting upregulated genes in red and downregulated genes in blue (*P*  <  0.05, ≥1 log_2_ fold change; *n*  =  3). (**C**) Venn-diagram showing overlapping and individual genes that are differentially expressed in both comparison pairs (upregulated in red, downregulated in blue).

Overall, the presence of CovS^Ala318Val^ had a very limited effect on the known CovRS regulon (24–27) (Fig. 3A through C). In total, eight genes were commonly differentially regulated in the mutant strains compared to their respective wild-type strains (Fig. 3C; Tables 2 and 3). As expected, *slo* expression was significantly upregulated in 5448^CovS:Ala318Val^ (fold change = 6.7) and SP1380^CovS:Ala318Val^ (fold change = 5.7). Our analysis revealed that this upregulation encompassed the entire *slo* operon, which includes *slo* and the genes encoding NADase (*nga*) and the NADase inhibitor (*ifs*). In addition, the genes encoding the known GAS virulence factors SIC (72), Ska (73), and SpyA (74), as well as the endonuclease Cas1c, were all upregulated in the presence of CovS^Ala318Val^.

**TABLE 2:** Differentially regulated genes in 5448^CovS:Ala318Val^ versus 5448^*a*^*^,^*^*b*^

	Locus tag	Gene	LogCPM	Log_2_FC	Adjusted *P* value
Upregulated	SP5448_RS08255	*scl-1*	3.204	2.987	0.005971
	SP5448_RS00855	*slo*	8.578	2.79	0.003042
	SP5448_RS00850	*ifs*	6.851	2.658	0.001854
	SP5448_RS00845	*nga*	7.468	2.606	0.003258
	SP5448_RS08240	*ska*	8.914	2.34	0.001854
	SP5448_RS08400	*sic*	11.28	2.259	0.003374
	SP5448_RS07500	*scpC*	6.966	2.042	0.01206
	SP5448_RS07465	*spyA*	6.714	1.826	0.001854
	SP5448_RS08385	*fbaA*	10.31	1.766	0.09903
	SP5448_RS02370	*sda1*	7.026	1.751	0.006559
	SP5448_RS08390	*scpA/B*	11.37	1.723	0.1361
	SP5448_RS03035	*cas1c*	5.95	1.512	0.003593
	SP5448_RS03025	*cas7c*	4.874	1.51	0.02349
	SP5448_RS03030	*cas4*	5.221	1.484	0.01273
	SP5448_RS03040	*cas2*	4.395	1.475	0.03059
	SP5448_RS03020	*cas8c*	5.886	1.324	0.02878
	SP5448_RS03090	*arcA*	7.377	1.269	0.02349
	SP5448_RS03095	GNAT family N-acetyltransferase	6.044	1.154	0.03867
	SP5448_RS03100	*argF*	6.623	1.119	0.05192
Downregulated	SP5448_RS00875	Cystathionine β-lyase (“*metC”*)	6.812	−1.1	0.02754
	SP5448_RS03720	Unknown	5.504	−1.242	0.03858
	SP5448_RS03725	Unknown	5.065	−1.426	0.02032
	SP5448_RS03730	Putative serine hydrolase	7.061	−1.516	0.007941
	SP5448_RS03910	*grab*	8.689	−2.126	0.03867

^*a*^ Statistically significant values (*P* < 0.05) highlighted in gray.

^*b*^ CPM, counts per million; FC, fold change.

**TABLE 3:** Differentially regulated genes in SP1380^CovS:Ala318Val^ versus SP1380^*a*^*^,^*^*b*^

	Locus tag	Gene	LogCPM	Log_2_FC	Adjusted *P* value
Upregulated	H7X56_08785	*sic*	10.98	2.604	0.0003881
	H7X56_01225	*scl-1*	3.37	2.511	0.1156
	H7X56_00915	*slo*	8.655	2.495	0.003976
	H7X56_08770	*fbaA*	10.38	2.374	0.03821
	H7X56_08775	*scpA/B*	11.33	2.306	0.04943
	H7X56_01240	*ska*	8.731	2.254	0.01948
	H7X56_00905	*nga*	7.4	2.197	0.005806
	H7X56_01990	*spyA*	6.64	2.182	0.003976
	H7X56_00910	*ifs*	6.926	2.165	0.005806
	H7X56_05845	*sda3*	8.099	1.985	0.04237
	H7X56_05850	Unknown	6.491	1.944	0.08315
	H7X 56_01960	*scpC*	6.812	1.911	0.1862
	H7X56_07110	*sda1*	7.161	1.722	0.07501
	H7X56_00920	Unknown	2.235	1.191	0.38264
Downregulated	H7X56_05735	Unknown	5.53	−1.278	0.04237
	H7X56_05725	Putative serine hydrolase	7.105	−1.364	0.04237
	H7X56_05555	*grab*	8.312	−2.431	0.05769

^*a*^ Statistically significant values (*P* < 0.05) highlighted in gray.

^*b*^ CPM, counts per million; FC, fold change.

Only 21 genes were differentially expressed in 5448^CovS:Ala318Val^ (Fig. 3A), and 13 genes were differentially expressed in SP1380^CovS:Ala318Val^ (Fig. 3B) compared to their respective parental strains. Genes that were significantly upregulated in 5448^CovS:Ala318Val^ compared to 5448 included several virulence factors known to be regulated by CovRS, including *sda1* (Sda1) (75), *scpC* (SpyCEP) (76), *arcA* (ADI) (77) and streptococcal collagen‐like protein 1 (*scl-1*) (78), and several genes of the *cas* operon (CRISPR-associated proteins). In SP1380^CovS:Ala318Val^, *scpA/B* (C5a peptidase) (79), *fbaA* (fibronectin-binding protein of group A streptococci) (80), and *sda3* (encoding a putative DNase) were significantly upregulated compared to SP1380. Although not statistically significant, upregulated *sda1*, *scpC* and *scl-1* expression was also observed in SP1380^CovS:Ala318Val^, indicating a similar trend compared to 5448^CovS:Ala318Val^. In contrast, only a small number of genes were downregulated in the presence of CovS^Ala318Val^, including a putative serine hydrolase encoded by *SP5448_RS03730* and *H7X56_05725* in 5448^CovS:Ala318Val^ and SP1380^CovS:Ala318Val^, respectively. The gene encoding the G-related alpha2M-binding protein (*grab*) (81) was downregulated in both 5448^CovS:Ala318Val^ and SP1380^CovS:Ala318Val^, though the change in SP1380^CovS:Ala318Val^ was not statistically significant.

In summary, introduction of CovS^Ala318Val^ into 5448 and SP1380 altered the known CovRS regulon by upregulating expression of a limited number of CovRS-regulated virulence genes, including the *nga-ifs-slo* operon, *ska* and *sic*, without affecting expression of other virulence factors known to be regulated by CovRS, such as *speB* or *hasA* (24–27).

### SP1450 triggers enhanced IL-1β secretion in THP-1 macrophages

**Fig 4:**
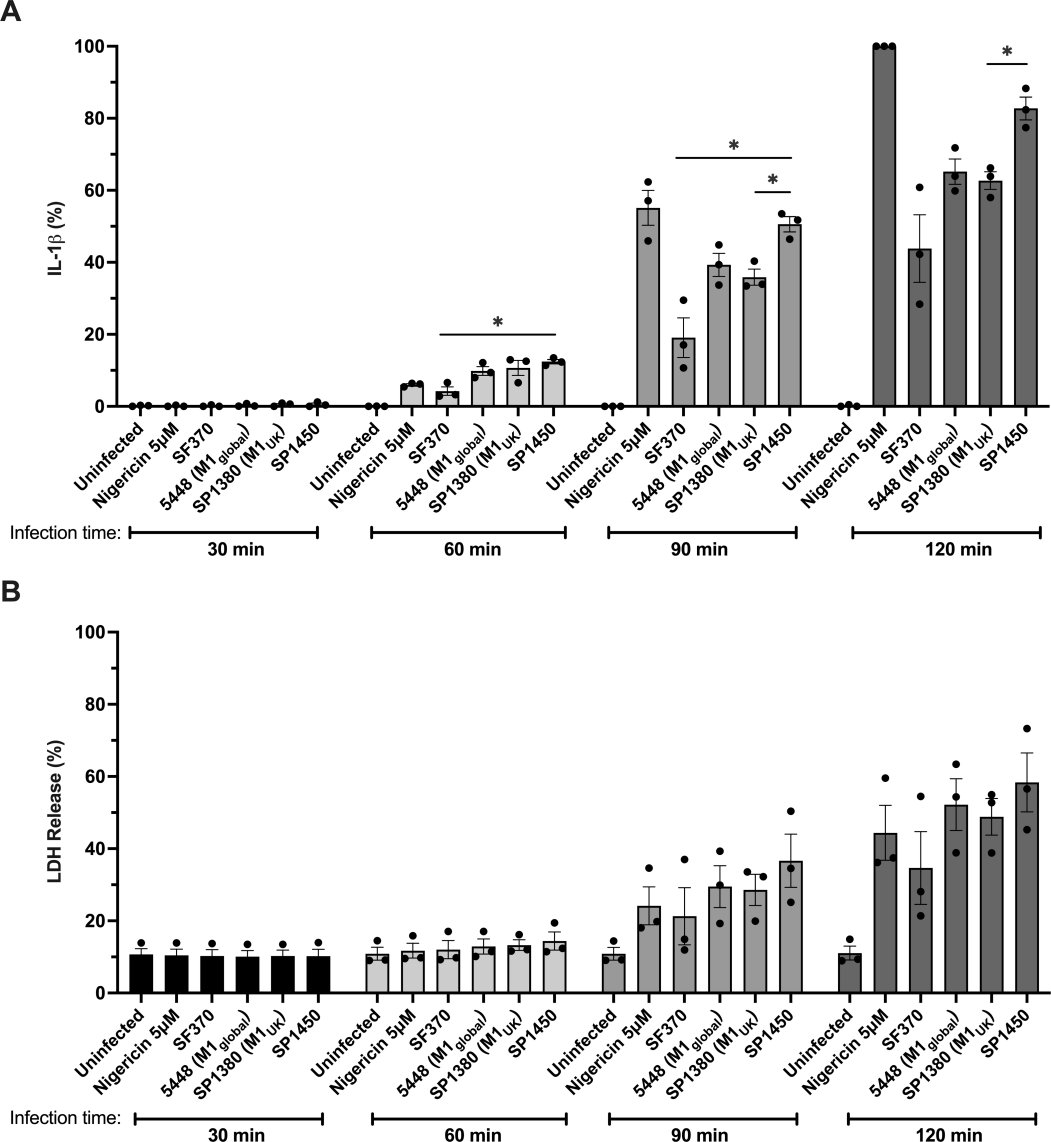
SP1450 triggers enhanced IL-1β secretion from differentiated THP-1 macrophages. Cells were infected with M1 GAS strains at MOI = 25. Nigericin, a potent bacterial ionophore, was used as a positive control for inflammasome activation. At the indicated timepoints, assay supernatants were collected and analyzed for (**A**) IL-1β and (**B**) LDH release. Data are presented as the mean percentage of the 2 h nigericin control for IL-1β release (**A**) or as mean percentage of complete cell lysis induced by Triton X-100 for LDH release (**B**) ± SEM of *n* = 3 independent replicate experiments. Significance was calculated using Brown-Forsythe and Welch analysis of variance tests. *P < 0.05.

The secretion of pro-inflammatory cytokines, such as IL-1β, plays a critical role during invasive GAS disease progression (47, 48), and it has been shown that *covRS*-mutated M1_global_ GAS triggers enhanced IL-1β production in human neutrophils (51). We and others have previously reported that SLO is the major driver of inflammasome-dependent IL-1β secretion in human macrophages (55–57). To test if the CovS^Ala318Val^ mutation, which results in increased SLO expression, might induce amplified inflammasome activation, human macrophage-like THP-1 cells were infected with SF370 (M1), 5448 (M1_global_), SP1380 (M1_UK_), and SP1450 (see Fig. 1) at a multiplicity of infection (MOI) of 25. At each timepoint, samples of the cell supernatants were taken to determine IL-1β levels in cell culture supernatants (Fig. 4A). As a positive control, cells were treated with 5 µM nigericin, an ionophore and established NLRP3 inflammasome activator (82). Since inflammasome activity can induce pyroptosis, we also assessed the levels of cytotoxicity by measuring the release of intracellular lactate dehydrogenase (LDH; Fig. 4B). While neither IL-1β nor LDH release was detected in cell culture supernatants at 30 min post-infection, IL-1β and LDH levels steadily increased in all samples with longer infection times and to varying degrees. All tested M1 strains induced higher levels of IL-1β than the nigericin control 60 min post-infection, with the exception of SF370. In contrast, only a minor increase of LDH release was detected at this timepoint, suggesting that inflammasome activation had not yet resulted in pyroptotic cell death. After 90 min of infection, the levels of secreted IL-1β in cells infected with SP1450 reached 91.7% of the respective nigericin control, which was significantly higher than samples infected with SF370 (34.6%) and SP1380 (65.1%), and markedly higher than 5448-infected samples (71.3%). At 120 min post-infection, levels of IL-1β were significantly higher in cells infected with SP1450 compared to SP1380 (82.7% and 62.7% of the nigericin control, respectively). No significant difference in IL-1β secretion was observed between 5448 and SP1380 at these timepoints, indicating that the M1_UK_ lineage-defining SNPs do not affect inflammasome activation under these conditions. Although the levels of LDH released by cells infected with SP1450 reached up to 58.4% of the total lysis control after 120 min, no significant difference between strains could be observed during the infection time course.

Taken together, these results show that the additional SNPs present in SP1450, including CovS^Ala318Val^, contribute to enhanced inflammasome-dependent inflammation elicited during GAS infection.

## DISCUSSION

Recent outbreaks of scarlet fever and an increase in invasive GAS infections in various regions worldwide (15, 19, 21, 83–85) have been attributed to several factors, including a dampened immunity to GAS following coronavirus disease 2019 restrictions (19) and the emergence of new virulent clones, notably M1_UK_ (10–13, 22). Over the past decade, M1_UK_ has spread rapidly and has become the dominant M1 lineage in several countries, including the UK, the Netherlands, and Australia (10, 13, 20, 22).

Differential gene expression and *in vivo* virulence of GAS are modulated by regulatory systems, including the CovRS two-component regulatory system that acts primarily through gene repression (34) and enables GAS to respond to environmental changes (28–32). In invasive infections caused by M1_global_ GAS, spontaneously occurring mutations in *covS* can enhance the pathogen’s virulence and drive disease progression (35–38, 44, 45). The “classic” CovRS regulon was identified through studies of mutants in which CovS kinase activity had been completely inactivated (24–27), a state than can result from larger deletions and frameshift mutations.

*covRS* mutants have been observed in the M1_UK_ lineage (23) but have not been fully characterized. In this study, we identified a novel mutation in the *covS* gene in three invasive Australian M1_UK_ isolates and investigated its impact on GAS virulence gene expression. Our findings revealed that the CovS^Ala318Val^ mutation increased expression of a subset of virulence genes typically upregulated in CovS-inactive mutants, such as *slo*, *ska*, and s*pyA*, but, unexpectedly, not others including *speA*, *hasA* (26, 27), or *pepO*, the latter of which has been identified as a marker for CovRS defects (39). In addition, CovS^Ala318Val^ did not affect *speB* expression, which is often lost in CovS-inactive mutants (25, 35, 37–39). Together, these observations suggest that the CovS^Ala318Val^ amino acid substitution does not completely inactivate CovS function but rather modulates its activity. Residue Ala318 is located in the HisKA domain of CovS (Fig. S3A). An early study reported that alanine has a significantly higher helical propensity than valine, and alanine-to-valine substitutions can affect protein function, particularly when they occur in the middle of an α-helix (86), as is the case for CovS^Ala318Val^ (Fig. S3B).

It was previously demonstrated that SpeB contributes to GAS colonization by impairing autophagy in epithelial cells, thereby facilitating intracellular GAS replication (87), and degrading proteins essential for the integrity of intercellular junctions (88, 89). In addition, SpeB also promotes GAS survival in subcutaneous (47) and intranasal (52) infection models. The limited impact of CovS^Ala318Val^ on *speB* expression suggests that M1_UK_ isolates with this mutation may retain their ability to colonize and persist in non-invasive settings. Moreover, the identification of the CovS^Ala318Val^ mutation in three separate invasive isolates could indicate that not all of the gene expression changes observed in CovS-inactive mutants are required to confer a fitness advantage during invasive infection. Instead, a mutation that partially preserves CovS function and thus CovR phosphorylation could have a similar impact. A recent study investigating CovR binding sites noted that while varying phosphorylation levels of CovR can impact its promotor binding affinity, there is no linear relationship between CovR occupancy and transcript-level changes (27). This suggests that, in addition to CovS, other co-factors may be required to regulate genes targeted by CovR. Indeed, the existence of intermediate regulators of the CovRS regulon has been reported (26); however, the mechanisms underlying this indirect regulation remain to be characterized. Continued longitudinal surveillance and comprehensive analyses of clinical CovRS-mutated isolates are warranted to fully elucidate the impact of *covRS* point mutations on GAS virulence and persistence.

Nasopharyngeal GAS colonization has been shown to be promoted by IL-1β signaling (52). As a potential explanation for this observation, it was suggested that GAS can survive the consequences of IL-1β signaling, particularly neutrophil activation, whereas commensal bacteria do not, allowing GAS to colonize their niche (52). In addition, *covRS*-mutated GAS has an increased ability to evade neutrophil clearance during invasive infection (36, 51). GAS-induced IL-1β signaling has previously been examined in the THP-1 model of human macrophages (90). Our earlier research demonstrated that the streptolysins SLO and SLS are primary activators of IL-1β production in this model (57). Here, we observed that the Australian M1_UK_ isolate SP1450, which carries the CovS^Ala318Val^ mutation and overexpresses SLO, triggers enhanced IL-1β secretion from human THP-1 macrophages compared to other invasive M1 strains. While this result suggests that CovS^Ala318Val^, responsible for increased SLO expression in SP1450, is the primary cause of the amplified IL-1β-dependent inflammation, the overall contribution of inflammasome signaling to M1_UK_ virulence remains to be determined.

In conclusion, this study characterizes a novel *covS* mutation detected in a subset of invasive Australian M1_UK_ isolates that alters the established CovRS regulon and potentially offers a fitness advantage during human infection, thereby providing new insights into the fine-tuning of CovS function and virulence strategies in M1_UK_.

## MATERIALS AND METHODS

### Bacterial strains and culturing conditions

All wild-type bacterial strains and isolates used in this study are listed in Table 4. The complete genome sequences of SP1380 (accession number CP060269) and SP1450 (accession number CP060266.1) were previously determined by Oxford Nanopore sequencing (22). All GAS strains were routinely streak-purified from cryo-stocks and grown on 5% horse blood agar (bioMérieux, Australia) at 37°C. Liquid cultures of GAS were grown in Todd-Hewitt broth (BD, USA) supplemented with 1% yeast (THY; Merck, USA) or in a chemically defined medium (91) to the indicated optical density at 600  nm (OD_600_). The *Escherichia coli* strain MC1061, which was used for site-directed mutagenesis, was grown in Luria-Bertani medium. For plasmid selection and maintenance, 100 µg/mL spectinomycin was added to both GAS and *E. coli* cultures.

**TABLE 4:** Bacterial strains and isolates used in this study

Strain	Description	Year of isolation	Reference
*E. coli* MC1061	Laboratory cloning strain	1980	(92)
*Streptococcus pyogenes*			
SF370	M1 wound infection isolate	1985	(65)
5448	Invasive M1T1/M1_global_ strain	Between 1994 and 1996	(66)
SP1380	Invasive M1_UK_ isolate	2019	(22)
SP1450	Invasive M1_UK_ isolate with CovS^Ala318Val^	2017	(22)
SP1466	Invasive M1_UK_ isolate with CovS^Ala318Val^	2018	(22)

### GAS mutant construction

All isogenic mutants created in this study are listed in Table 5. Isogenic mutants of 5448, SP1380, and SP1450 were generated using a previously published protocol for creating markerless isogenic GAS mutants (93). PCR primers were designed to replace the *covS*, *rofA*, and *rocA* genes in SP1450 with the respective genes from 5448 (M1_global_) and to introduce the *covS* gene from SP1450 (including the *covS*^C953T^ SNP, which results in the CovS^Ala318Val^ mutation) into 5448 and SP1380. All PCR primer sequences used for the creation of isogenic GAS mutants are provided in Table S1. Successful gene replacements were confirmed via PCR and DNA sequencing (Genetic Research Services, University of Queensland, Australia) using the primers specified in Table S1.

**TABLE 5:** Isogenic GAS mutants used in this study

Strain	Description	Reference
5448^CovS:Ala318Val^	Isogenic 5448 mutant with introduced CovS^Ala318Val^	This study
SP1380^CovS:Ala318Val^	Isogenic SP1380 mutant with introduced CovS^Ala318Val^	This study
SP1450^covS*^	Isogenic SP1450 mutant with M1_global_ *covS* sequence	This study

### Precipitation of bacterial supernatant proteins and immunoblotting

Total protein from bacterial culture supernatants was isolated as previously described (91). Briefly, supernatants of GAS cultures grown in THY were strained through a 0.22 µm filter (Merck; SLGP033RS) and combined with concentrated trichloroacetic acid (10% of the final volume, purchased from Sigma-Aldrich, USA). Total supernatant protein was precipitated overnight at 4°C, harvested by centrifugation and washed in ice-cold pure ethanol. Dried protein pellets were resuspended in a loading buffer containing 100 mM dithiothreitol (Sigma-Aldrich, D0632) and boiled for 15 min with occasional vortexing. Samples were separated by SDS-PAGE before being transferred onto methanol-activated Immobilon polyvinylidene difluoride membranes (Merck, IPFL00010) using a standard wet transfer system (Bio-Rad, USA). Membranes were blocked in Intercept Blocking Buffer (LI-COR Biosciences, 927-70003) for 1 h at room temperature and then incubated overnight at 4°C with primary antibodies targeting SLO (91) and SpeB (Toxin Technology, USA; PBI222) at a 1:1,000 dilution. Membranes were incubated with fluorescent secondary antibodies (DyLight 800 anti-mouse or anti-rabbit IgG (H + L); New England Biolabs, USA; used at 1:10,000 dilution) for 60 min at room temperature before proteins were detected by scanning the membranes with an Odyssey Imaging System (LI-COR Biosciences).

### RNA isolation

GAS cell pellets from THY liquid cultures grown to the mid-exponential growth phase (OD_600_ = 0.4) were resuspended in 700 µL RLT buffer (Qiagen, Germany) supplemented with 1% β-mercaptoethanol (Bio-Rad). Samples were transferred into lysing matrix B tubes (MP Biomedicals, USA) and lysed using a FastPrep homogenizer (MP Biomedicals) for 2× cycles of 30 s at a speed of 9 m/s and with a 3-min pause between cycles. Cell debris was removed by centrifugation at 16,000 × *g* for 1 min. RNA was immediately extracted using the RNeasy Mini Kit following the manufacturer’s protocol (Qiagen), which included an on-column digestion with DNase I (Qiagen) to remove excess DNA from the samples. Final RNA was diluted in nuclease-free water, and concentrations were determined with a NanoDrop spectrophotometer (Thermo Fisher Scientific, USA). Following the extraction, a total amount of 5 µg RNA was used for an additional, off-column DNA digestion using the TurboDNase kit according to the manufacturer’s instructions (Thermo Fisher Scientific). All samples were confirmed DNA-free by KAPA HiFi PCR (Roche, Switzerland). High RNA integrity (RIN > 7) was confirmed using the TapeStation platform (Agilent Life Sciences and Chemical Analysis, USA).

### Real-time PCR

For real-time PCR (RT-PCR) assays, 500 ng RNA was transcribed into cDNA using the GoScript Reverse Transcriptase kit according to the manufacturer’s instructions (Promega, USA). Diluted cDNA was amplified using the QuantiNova SYBR Green PCR kit (Qiagen). All primers used for RT-PCR are listed in Table S2. The relative expression of target genes was determined using standard “fast reaction” parameters on the QuantStudio6 System (Thermo Fisher Scientific); an initial denaturation cycle (95°C for 5 min) was followed by 45 cycles of amplification (95°C for 10 s, 60°C for 10 s, and 72°C for 10 s). Following the PCR, melting curves were generated to confirm that only a single reaction product was amplified for each primer pair (data not shown). Relative mRNA transcript levels of the target genes were determined using the ΔΔCt method (94) and normalized to reference gene *gyrA* (95).

### RNA sequencing analysis

Ribosome depletion, Illumina library prep, and single-end RNA sequencing with NovaSeq was undertaken by The Australian Genome Research Facility (Melbourne, Australia). Raw sequence data were analyzed with tools available on Galaxy Australia (96). Default parameters were used for all tools unless stated otherwise. For quality control, sequence data were checked with FastQC (Galaxy, version 0.73) (97). Sequence reads were then mapped to the respective reference genomes using the Bowtie2 tool (Galaxy, version 2.5.0) with the parameter “very sensitive local” (98). Reads that map to multiple locations were filtered, and the number of mapped reads per gene was calculated with featureCounts (version 1.6.4) (99), with the minimum overlap length set to 20 bp. Differential gene expression analysis in the comparison pairs 5448/5448^CovS:Ala318Val^ and SP1380/SP1380^CovS:Ala318Val^ was performed using limma-voom (Galaxy, version 3.50.1) (100, 101). Weights were applied to outliers to decrease the weight of variable samples. Genes with a count per million of <1 in less than four samples per comparison pair were not included. Genes were normalized using the trimmed mean of *M* values method. Genes with a log2 fold change value of ≥1 and ≤−1 and an adjusted *P* value of ≤0.05 were considered significant (102). Data were visualized with Glimma (version 1.10.0) (103). Multidimensional scaling analysis visualizing replicate clustering is provided in Fig. S4.

### Infection dose preparation for *in vitro* inflammasome assays

For preparation of infection doses used for *in vitro* macrophage assays, liquid bacterial cultures were grown statically in a chemically defined medium (91) to the mid-logarithmic growth phase at OD_600_ = 0.3. The cultures were collected by centrifugation for 5 min at 8,000 × *g* and washed in Dulbecco’s phosphate-buffered saline (Thermo Fisher Scientific). Bacterial cell pellets were then resuspended and diluted in Roswell Park Memorial Institute (RPMI) 1640 (Thermo Fisher Scientific) supplemented with 2% fetal bovine serum (Bovogen, Australia) to the indicated MOI.

### Culture and differentiation of THP-1 cells

Human THP-1 monocytic cells expressing Cas9 (104) were maintained in RPMI medium supplemented with 10% fetal bovine serum (Bovogen), 10 mM HEPES (Sigma-Aldrich), and 1 mM sodium pyruvate (Thermo Fisher Scientific). The THP-1 cells were differentiated into a macrophage-like phenotype using phorbol myristate acetate (Sigma-Aldrich) as previously described (57).

### Macrophage infection assay

Differentiated THP-1 cells were primed for inflammasome activation with lipopolysaccharide as previously described (57). Following priming, the cell medium was removed and replaced with the indicated treatments or GAS infection dose diluted in assay medium. Cells treated with assay medium only served as a negative control, while the NLRP3 inflammasome-activating ionophore nigericin (Sigma-Aldrich) was used as a positive control. Cell culture plates were centrifuged for 10 min at 500 × *g* to bring bacteria into contact with the cells, before being placed back into the incubator for the indicated time periods. Following the assay, plates were centrifuged for 5 min at 300 *× g* to settle debris. Finally, assay supernatants were collected and probed for the secretion of LDH and IL-1β as previously described (57).

### Statistical analysis

All statistical analyses were carried out using GraphPad Prism (versions 9 and 10). Data shown were collected from at least three independent experiments and are represented as mean ± standard error of the mean unless indicated otherwise. Significance was calculated using either ordinary or Brown-Forsythe and Welch one-way analysis of variance in combination with Dunnett or Tukey multiple comparisons post hoc tests, or Welch *t*-tests, as indicated. *P* values of <0.05 were considered statistically significant.

## Data Availability

The RNA sequencing reads and associated gene expression profiles have been deposited in the National Center for Biotechnology Information Gene Expression Omnibus database under the accession number GSE280498.

## SUPPLEMENTAL MATERIAL

Supplemental material mbio.03366-24-s0001.docx Supplemental figures and tables.

ASM does not own the copyrights to Supplemental Material that may be linked to, or accessed through, an article. The authors have granted ASM a non-exclusive, world-wide license to publish the Supplemental Material files. Please contact the corresponding author directly for reuse.
