# Supplementary material for: Characterization of a novel covS SNP identified in Australian group A Streptococcus isolates derived from the M1UK lineage
Source: mBio. 2024 Dec 17;16(2):e03366-24. doi: 10.1128/mbio.03366-24 (PMC11796353; doi:10.1128/mbio.03366-24)
Supplement: Supplemental material — Supplemental figures and tables. [file mbio.03366-24-s0001.docx]

**Supplemental Figures**

**Figure S1.**

**CovS^Ala318Val^ does not affect production of mature SpeB**. Strains were grown for 48 hours in THY liquid culture to the stationary growth phase (OD_600_ ≥ 1.2). Total supernatant protein was harvested using TCA precipitation, and SpeB was visualized via immunoblotting (n = 1).

**Figure S2.**

**CovS^Ala318Val^ does not affect bacterial growth in THY**. Liquid cultures were grown overnight in a 96-well plate reader at 37˚C. The OD_600_ was measured every 20 minutes for 8 hours (n = 1). Error bars represent standard deviation.

**Figure S3.**

**CovS^Ala318Val^ is located in the HisKA domain. (A)**CovS amino acid sequence of GAS 5448 with putative conserved and functionally important domains: TM1 and TM2, transmembrane helixes 1 and 2; HAMP, present in histidine kinases, adenylate cyclases, methyl-accepting proteins, and phosphatases; HisKA, histidine kinase domain; HATPase, histidine-kinase like ATPase. The Ala318 residue (highlighted in red), which is mutated in SP1450 and SP1466, is located in the HisKA domain. **(B)** 3D structure prediction model of M1_global_ wildtype CovS created with AlphaFold. HisKA domain highlighted in blue, Ala318 residue highlighted in red. The per-residue confidence score (pLDDT) for all residues in this domain lies between 70 – 90 (Confident). The predicted protein sequence data was obtained from the Japanese M1T1 isolate 476 (1), UniProt ID: J7M179, and colorized with PyMOL software.

**Figure S4.**

**Multidimensional scaling (MDS) plots of the transcriptional variation** in **(A)** 5448 and 5448^CovS:Ala318Val^ and **(B)** SP1380 and SP1380^CovS:Ala318Val^. To account for possible RNA degradation at later growth stages, strains were grown in THY liquid culture to the mid-exponential growth phase (OD_600_ = 0.4). Data points correspond to individual biological replicates.

**Supplemental Tables**

| **Table S1.** Primers used for GAS mutant construction | |  |
| --- | --- | --- |
| **Primer name** | **Primer sequence (5’**→ **3’)** | **References** |
| rofA_fwd | GTCGTCAGACTGATGGGCCCTTCTTTAAATTAAAGCAATAAACTTG | (2) |
| rofA_rev | CATAACCTGAAGGAAGATCTGCTCTGATTCGGTTAAGTAG | (2) |
| rofA_seq_fwd | TATTTTTAGCTTAGATAGAATTGAAA | (2) |
| rofA_seq_rev | TTGTATTGACAGGATTGCTTG | (2) |
| rocA_fwd | GTCGTCAGACTGATGGGCCCTAGTAGGGATGTCAAAAAC | this study |
| rocA_rev | CATAACCTGAAGGAAGATCTGCCTCAACGAATATGAGATC | this study |
| rocA_seq_fwd | TGACAGAACTTATGATAAAATAAAGA | this study |
| rocA_seq_rev | GCTTGGAAGAAACAATAAACTATT | this study |
| covS_fwd | TGATGGGCCCTGCCCGTATTCGTGCTATTTTC | this study |
| covS_rev | AGGAAGATCTGAACTTCTTGTTCAATACGAGAAATG | this study |
| covS_seq_fwd | CATATCGAAGATCAGTATCGTGGG | this study |
| covS_seq_rev | TAATGATTACATACTATACCTGTC | this study |
| slo_5'UTR_fw | GTCGTCAGACTGATGGGCCCATATGTATAAGGTGCCAAAG | this study |
| slo_5'UTR_rv | TCGAACCATATTTAAATGTTTTTTTGTTAGACATG | this study |
| slo_3'UTR_fw | AACATTTAAATATGGTTCGATTACTTATAAGTAG | this study |
| slo_3'UTR_rv | CATAACCTGAAGGAAGATCTTAGTCTCTCTGATATCACTAG | this study |
| slo_seq_F1 | ACCGTCAAAGCATACTAGC | this study |
| slo_seq_R1 | TAGCTTCAAGGTCACGATG | this study |

| **Table S2.** RT-PCR primers used in this study | | |
| --- | --- | --- |
| **Primer name** | **Primer sequence (5’**→ **3’)** | **References** |
| slo_F | CAAAGCAACGGTTGAGGTCA | (3) |
| slo_R | GCAGGAAGCGTATTACCACC | (3) |
| speB_F | TGCTGACGGACGTAACTTCT | (4) |
| speB_R | CCACCAGTACCAAGAGCTGA | (4) |
| gyrA_F | CGACTTGTCTGAACGCCAAA | (4) |
| gyrA_R | GTCAGCAATCAAGGCCAACA | (4) |

**Supplemental References**

1. Miyoshi-Akiyama T, Zhao J, Kikuchi K, Kato H, Suzuki R, Endoh M, Uchiyama T. 2003. Quantitative and qualitative comparison of virulence traits, including murine lethality, among different M types of group A streptococci. J Infect Dis 187:1876-1887.

2. Davies MR, Keller N, Brouwer S, Jespersen MG, Cork AJ, Hayes AJ, Pitt ME, De Oliveira DMP, Harbison-Price N, Bertolla OM, Mediati DG, Curren BF, Taiaroa G, Lacey JA, Smith HV, Fang N-X, Coin LJM, Stevens K, Tong SYC, Sanderson-Smith M, Tree JJ, Irwin AD, Grimwood K, Howden BP, Jennison AV, Walker MJ. 2023. Detection of *Streptococcus pyogenes* M1_UK_ in Australia and characterization of the mutation driving enhanced expression of superantigen SpeA. Nat Commun 14:1051.

3. Brouwer S, Jespersen Magnus G, Ong Cheryl-lynn Y, De Oliveira David MP, Keller B, Cork Amanda J, Djoko Karrera Y, Davies Mark R, Walker Mark J. 2022. *Streptococcus pyogenes* hijacks host glutathione for growth and innate immune evasion. mBio 13:e00676-22.

4. Brouwer S, Cork Amanda J, Ong Cheryl-Lynn Y, Barnett Timothy C, West Nicholas P, McIver Kevin S, Walker Mark J. 2018. Endopeptidase PepO regulates the SpeB cysteine protease and is essential for the virulence of invasive M1T1 *Streptococcus pyogenes*. J Bacteriol 200:e00654-17.
